# Inequalities in NHS staff support among those from ethnic minority and migrant groups during the COVID-19 pandemic

**DOI:** 10.1136/oemed-2025-110203

**Published:** 2026-04-07

**Authors:** Bethany Croak, Danielle Lamb, Sharon A M Stevelink, Rupa Bhundia, Juliana Onwumere, Brendan Dempsey, Pamela Almeida-Meza, Zoe Chui, Neil Greenberg, Rosalind Raine, Charlotte Woodhead, Stephani L Hatch, Rebecca Rhead

**Affiliations:** 1 Department of Psychological Medicine, King’s College London, London, UK; 2 Department of Primary Care and Population Health, University College London, London, UK; 3 NIHR ARC North Thames, University College London, London, UK; 4 King’s Centre for Military Health Research, King’s College London, London, UK; 5 Bethlem Royal Hospital, South London and Maudsley NHS Foundation Trust, London, UK; 6 ESRC Centre for Society and Mental Health, King’s College London, London, UK

**Keywords:** COVID-19, Mental Health, Health Personnel, Occupational Health

## Abstract

**Objectives:**

During the COVID-19 pandemic, National Health Service staff support services were implemented to promote healthcare workers’ (HCWs) well-being, alongside informal support from colleagues and managers. Certain groups may be less likely to access support, including HCWs from ethnic minority and migrant groups. These HCWs are more likely to experience discrimination and abuse at work, which may also erode access to positive and protective resources. Therefore, this study examined variation in formal support programme use and perceptions of support from managers and colleagues by ethnicity and migration status.

**Methods:**

This study analysed survey data from 9769 HCWs in England who completed the baseline survey (launched April 2020) and the 6-month follow-up using descriptive statistics and binary logistic regression.

**Results:**

At 6 months, 51% of participants met the threshold for probable common mental disorder. HCWs from White Other (Adjusted Odds Ratio (AOR) 0.79; 95% CI 0.64 to 0.99) and Asian ethnic groups (AOR 0.65; 95% CI 0.57 to 0.74) were less likely to feel supported by their colleagues than White British HCWs. Similarly, those born outside of the UK and European Union were less likely to feel supported by their colleagues than UK-born HCWs (AOR 0.70; 95% CI 0.52 to 0.94). No variations in support programme use or manager support were found across ethnicity or migration status.

**Conclusions:**

The study suggests equitable formal support but identified critical disparities in perceived support from colleagues for HCWs during the COVID-19 pandemic. Improving workplace well-being should address the underlying social and structural factors that influence peer support and belonging.

**WHAT IS ALREADY KNOWN ON THIS TOPIC**

- In the UK, healthcare workers (HCWs) from ethnic minority and migrant groups are more likely than White British HCWs to experience discrimination and abuse from colleagues. Such experiences may reduce trust in workplace support and undermine perceptions of support from managers and peers, but these outcomes have not previously been examined in large-scale quantitative research.

**WHAT THIS STUDY ADDS**

- The study indicates that formal support mechanisms for HCWs during the COVID-19 pandemic were generally equitable. However, it highlights significant disparities in perceived colleague support, with HCWs from some ethnic minority groups and HCWs born outside of the UK reporting lower levels of peer support compared with White British HCWs.

**HOW THIS STUDY MIGHT AFFECT RESEARCH, PRACTICE OR POLICY**

- These findings suggest that while structured support systems may be in place, the day-to-day experiences of workplace camaraderie and informal support vary considerably, underscoring the need for targeted interventions to foster a more inclusive and supportive work environment.

## Background

 During the early phases of the COVID-19 pandemic, 59% of healthcare workers (HCWs) in England met the threshold for a probable common mental disorder.^1^ In response, the National Health Service (NHS) introduced a range of support interventions at a national and Trust level, including Wellbeing Hubs^2^ (rapid access to free mental health support for HCWs) and practical support such as free parking.^3^ This was alongside existing informal support from managers and colleagues.

Formal support services were widely used early in the pandemic, with 58% of HCWs reporting engaging with at least one service.^4^ Practical support (eg, free parking) was generally valued, whereas psychological services were perceived as harder to access due to stigma and time pressures.^3^ There is also evidence that uptake and perceived relevance varied across groups. For example, use of NHS well-being Hubs was reportedly lower among Black and Asian HCWs, who expressed concerns that services were not equipped to address racism.^5^ More broadly, psychotherapy models have been criticised for reflecting Western, individualistic values, potentially limiting accessibility for people from diverse cultural backgrounds.^6^ Despite this emerging evidence, it remains unclear whether NHS staff support provision was equitably accessed across ethnic and migrant groups, as no large-scale quantitative study has examined support use by ethnicity and migration status.

Informal support from managers and colleagues has been associated with better mental health outcomes^7^ during the COVID-19 pandemic. However, support from managers and colleagues relies on working relationships, and these may not be evenly distributed. HCWs from ethnic minority groups consistently report higher levels of bullying, harassment and discrimination from colleagues and managers.^8^ For example, the most recent NHS Workforce Race Equality Standard report, which collects and monitors data across a series of key indicators of workforce equality, found that 17% of staff from an ethnic minority group reported discrimination from a manager or colleague, compared with 7% of White British staff.^9^ HCWs born outside the UK also face disadvantages. They are more likely than UK-born staff to face abuse from other colleagues,^10^ and international nurses from White ethnic groups report particularly high levels of abuse.^9^ Beyond this, migrant HCWs may face cultural and language barriers, less familiarity with support systems and greater job insecurity.^11^ Such experiences may undermine trust, belonging and willingness to seek support, potentially restricting access not only to formal services but also to everyday peer support, which together may restrict their ability to access workplace support.

While inequalities in negative workplace experiences are well documented, far less is known about whether these extend to positive and protective resources such as support from colleagues and managers, or to uptake of formal well-being services. Qualitative studies suggest this may be the case, with HCWs from ethnic minority groups reporting less support from managers during the COVID-19 pandemic, particularly when managers were White British.^12^ However, large-scale quantitative evidence is limited. Understanding whether disparities exist in support use and perceived support is important for evaluating the equity of staff well-being provision and has implications for well-being, staff sickness absence and retention.^13^

## Method

This study analysed data from a large-scale survey of HCW mental health and well-being (NHS CHECK) to answer the following research questions:

1. How does support programme uptake vary by ethnicity and migration status?
2. How does the perception of support from managers vary by ethnicity and migration status?
3. How does the perception of support from colleagues vary by ethnicity and migration status?

NHS CHECK is a longitudinal cohort study comprising online surveys and semi-structured interviews,^14^ investigating HCW well-being during the COVID-19 pandemic and beyond (April 2020–present). NHS employees (clinical and non-clinical) from 18 participating NHS trusts (online supplemental material 2) were eligible to take part (see Lamb *et al* for details).^1^

The baseline survey opened in April 2020 and closed in January 2021 (n=23 346; response rate 15%), with the second survey launched in October 2020 and closing in August 2021 (n=10 671; response rate 45.5%) (online supplemental material 3). Data from participants who had completed baseline (T1) and the 6-month follow-up (T2) were included in this study (n=9769). Of those who completed baseline, ~45% completed the 6-month follow-up. The surveys were split into a short and long version, whereby after completing the first set of questions, participants were given the choice to complete a longer survey. All measures used in this analysis are from the short survey and response frequencies refer to the short surveys.

### Measures

Demographic and occupational information, including ethnicity, migration status, role, age and sex, were collected at baseline. Ethnicity was captured using the 17 ethnicity categories from the 2011 UK Census (online supplemental material 4). Due to small numbers in some groups, the 17 detailed ethnicity categories were collapsed into five broader groups: White British, White Other, Black, Asian and Mixed/Other. While this approach inevitably reduces within-group diversity, it allows sufficient statistical power for analysis and provides more nuance than binary White/Black and ethnic minority categorisation. In particular, we analysed White British and White Other separately. For migration status, participants were asked to select one of three options, ‘born in UK’, ‘born in European Union (EU) (not UK)’ and ‘born elsewhere’. Participants were asked to specify their main role and this was combined into four role categories: doctor, nurse, other clinical and other non-clinical.

#### Support used

At the 6-month follow-up (T2), participants were asked whether they had used any national or local support services provided by NHS England or their Trust. The list of programmes, developed with NHS Trusts and the NHS CHECK PPI group, was grouped into seven categories by the research team (Occupational Health (OH), Psychiatry and mental health experts). Free-text responses were recoded into the same categories:

1. Formal mental health support (therapy via general practitioner, community team, well-being hub, private practice).
2. Employee assistance programmes (EAPs) (short-term counselling, referrals to mental health teams).
3. OH (multidisciplinary support for health problems linked to work).
4. well-being support (helplines, apps, charities, chaplains).
5. Workplace support (virtual staff rooms, mentoring, manager/team reflective practice).
6. Practical support (food, parking, accommodation, childcare, financial advice, relaxation rooms).
7. Information support (guidance on self-care, stress management, COVID-19).

Support types were analysed descriptively to provide an overview of the range of services accessed, even though there is little theoretical rationale to suggest inequitable access to some support services (eg, retail or practical discounts). For regression models, due to low numbers within some categories, a binary outcome was defined: ‘support used’ (if any programme was accessed) versus ‘no support used’.

#### Feeling supported

Participants were also asked: “Since the COVID-19 pandemic, how well do you feel supported by your manager?” and the same question about support from colleagues. Participants were asked to rate the extent to which they felt supported by their colleagues and by their manager on a 5-point scale anchored from: ‘not at all’, ‘a little bit’, ‘moderately’, ‘quite a bit’ to ‘extremely’.

Binary outcome variables were created. Participants who selected ‘not at all’ or ‘a little bit’ were categorised as little/no support, and those who selected ‘moderately’ or ‘quite a bit’ or ‘extremely’ were categorised as supported.

#### Mental health

The 12-item General Health Questionnaire (GHQ-12), issued at T2, was used to estimate the prevalence of probable common mental disorders, with a score of four or more indicating a probable common mental disorder, using the 0-0-1-1 scoring method.^15^ A threshold at this level has an estimated sensitivity of 69% and specificity of 88%.^16^ The GHQ-12 has good internal consistency (Cronbach’s α=0.94).^17^ GHQ-12 total score (T2) was used to adjust for mental health difficulties in all regression analyses as a preliminary regression showed that there was an association between support (use and perceptions) and psychological distress (GHQ-12 scores) at T2 (online supplemental material 5).

### Statistical analysis

Secondary data analysis was performed on NHS CHECK participants who had completed the baseline survey and the 6-month follow-up (n=9769). Analyses were conducted using Stata (V.17).^18^

We calculated the proportion of participants reporting support use and perceived support from managers or colleagues, each treated as a distinct outcome. Two-level random effects logistic regression models accounted for clustering by NHS Trusts. Intraclass correlation coefficients indicated modest clustering of outcomes by NHS Trust (7.6% support use; 2.1% manager support; 0.6% colleague support). Likelihood ratio tests confirmed that multilevel models fitted significantly better than single-level models (p<0.05). We also examined the distribution of random effects and found no evidence of deviation from normality.

Primary outcomes were support programme use (RQ1) and perceived support from managers and colleagues (RQ2-3). Ethnicity and migration status were the main exposures, with unadjusted and adjusted models for each outcome. Models (Adjusted Odds Ratios (AORs) reported) adjusted for age, sex, GHQ-12 score and role, given previous evidence that younger, female HCWs and those with greater psychological distress were more likely to use support services,^4^ and that nurses had poorer mental health than other HCWs.^1^

To improve representativeness and reduce potential non-response bias, response weights were generated using a raking algorithm for age, sex, role (clinical/non-clinical) and ethnicity based on Trust-level staff data. Reported frequencies are unweighted; proportions are weighted. Weighted proportions, therefore, provide estimates more closely reflecting the wider NHS workforce composition. This approach is commonly used in survey research where participation varies across key demographic groups.^19^

## Results

### Data completeness

Missing data for exposure, outcome and predictor variables is detailed in table 1; the common mental disorder variable is reported in online supplemental material 6. As the proportion of missing data on each individual predictor and outcome variables was no more than 5%, complete case analysis was deemed appropriate.^20^ For all adjusted regression models, the proportion of the sample included was ~90% of the total sample; specific numbers included in each analysis are provided below the regression tables (tables 2 and 4).

**Table 1:** Demographic characteristics and workplace experiences of participants (N=9769)

	Frequency (n)	Unweighted (%)	Weighted (%)
Exposure variables (T1)			
Ethnicity			
White British	7814	80.0	74.7
White other*	836	8.6	8.0
Black†	278	2.9	4.7
Asian‡	503	5.2	9.8
Mixed/other§	285	2.9	2.5
Missing	53	<1	<1
Migration status			
Born in UK	8269	84.7	80.6
Born in EU (not UK)	573	5.9	5.8
Born elsewhere	849	8.7	13.1
Missing	78	<1	<1
Outcome variables (T2)			
Support programme use			
Support used	8042	82.3	83.1
Support not used	1533	15.7	15.0
Missing	194	2.0	1.9
Felt supported by manager			
Little/no support	2803	28.7	30.8
Supported	6944	71.1	69.0
Missing	22	<1	<1
Felt supported by colleagues			
Little/no support	1830	18.7	18.6
Supported	7924	81.1	81.3
Missing	15	<1	<1
Covariates (T1)			
Age (T1)			
≤30	1452	14.9	16.3
31–40	1948	19.9	22.8
41–50	2566	26.3	24.3
51–60	2635	27.0	24.1
≥61	700	7.2	7.8
Missing	468	4.8	4.7
Sex (T1)			
Male	1780	18.2	24.1
Female	7911	81.0	75.2
Missing	78	<1	<1
Role (T1)			
Doctor	702	7.2	10.2
Nurse	2342	24.0	27.8
Other clinical	2833	29.0	31.7
Non-clinical	3775	38.6	29.6
Missing	117	1.2	<1

Percentages may not sum to 100 due to rounding.

* Includes White Irish, White Gypsy or Irish Traveller and any other White background.

† Includes Black African, Black Caribbean and any other Black background.

‡ Includes Indian, Pakistani, Bangladeshi, Chinese, Arab or any other Asian background.

§ Includes any mixed background or other ethnic groups.

EU, European Union.

**Table 2:** Variations in support programme use by ethnicity and migration status

	Support used (T2)	Unadjusted		Adjusted*	
	n (%)	OR	95% CI	AOR	95% CI
Ethnicity†					
White British	6437 (84.8)	–	–	–	–
White other‡	686 (84.4)	0.87	0.70 to 1.08	0.85	0.66 to 1.10
Black§	227 (84.3)	0.86	0.68 to 1.08	0.97	0.65 to 1.45
Asian¶	412 (84.7)	0.87	0.47 to 1.63	0.85	0.45 to 1.60
Mixed/other**	236 (87.2)	1.10	0.65 to 1.87	1.00	0.56 to 1.78
Migration status††					
Born in UK	6825 (84.2)	–	–	–	–
Born in EU (not UK)	471 (84.6)	1.10	0.72 to 1.68	0.97	0.62 to 1.52
Born elsewhere	681 (83.6)	0.81	0.62 to 1.06	0.85	0.63 to 1.14

* Model adjusted for age, sex, role and GHQ-12 score at T2 and reported as AOR.

† n=9447 included in the unadjusted analysis, n=8751 included in the adjusted analysis. Counts reflect participants with complete data on the exposure (ethnicity) and, for adjusted models, complete data on all covariates included in the model.

‡ Includes White Irish, White Gypsy or Irish Traveller and any other White background.

§ Includes Black African, Black Caribbean and any other Black background.

¶ Includes Indian, Pakistani, Bangladeshi, Chinese, Arab or any other Asian background.

** Includes any mixed background or other ethnic groups.

†† n=9421 included in the unadjusted analysis, n=8726 included in the adjusted analysis. Counts reflect participants with complete data on the exposure (migration status) and, for adjusted models, complete data on all covariates included in the model.

AOR, Adjusted Odds Ratio; EU, European Union; GHQ-12, 12-item General Health Questionnaire.

### Sociodemographic characteristics

In total, 9769 HCWs completed both the baseline and 6-month follow-up questionnaire (short version). The response rate for the ‘Mixed/Other’ group was similar to White British HCWs at 89%. Response rates for the other ethnic groups were lower: ‘Black’ (81%), ‘Asian’ (82%) and ‘White Other’ (88%). Similarly, the response rate for HCWs who were born in the EU (87%) and elsewhere (84%) was lower than those born in the UK (89%).

Within ethnic groups, HCWs from the White Other ethnic group had the highest proportion of participants from migrant groups (79%) (proportion of HCWs from migrant groups by ethnicity can be found in online supplemental material 7.

### Common mental disorders and support use

In all analyses, we adjusted for common mental disorder (GHQ-12 score). Over half of the participants met the cut-off (score of four or more) on the GHQ-12 at T2 (51%), with a median score of 4 and an IQR of 1 to 8, indicating that over half of the participants had symptoms of a probable common mental disorder (online supplemental material 6).

### Support programme use (variations in ethnicity and migration status)

In total, 8042 (83%) reported using one or more support programme (table 1). Table 2 and online supplemental material 8 show the proportion of staff who used support programmes by ethnicity and migration status. The multilevel binary logistic regression models (table 2) showed no evidence that programme use varied by ethnicity or migration status.

The most common were practical support (27.1%), OH (21.5%), therapy (21.2%) and information support (24.1%). Well-being support (9.2%), workplace support (10.3%) and EAPs (5.3%) were less frequently used (table 3).

**Table 3:** Type of support used at T2 across the whole sample (n=9769)

Support type (T2)	Used (n, %)
Formal mental health support	2173 (21.2)
Employee Assistance Programmes	572 (5.3)
Occupational health	2046 (21.5)
Practical support	2797 (27.1)
well-being support	823 (9.2)
Workplace support	1082 (10.3)
Information support	2275 (24.1)

By ethnicity (online supplemental material 8), therapy use was highest among White Other (25.1%) and lowest among Black staff (14.0%). EAP uptake was low overall but slightly higher among Black HCWs (6.6%). OH use was higher in Black (31.5%) and Mixed/Other (33.1%) groups than White British (20.5%). Practical support was broadly consistent (22%–28%). Workplace support was highest in Mixed/Other (15.6%) and Black (14.6%) groups, while information support ranged from 22.1% (Asian) to 24.4% (White British).

By migration status, therapy use was most common among EU staff (27.6%) compared with UK-born (21.9%) and Other non-EU (13.9%). EAP use was slightly higher among Other non-EU staff (5.5%). OH and workplace support were also somewhat higher in this group (24.6% and 13.5%), while information and well-being support were similar across groups (9%–10% and 24%–25%).

### Feeling supported (variations by ethnicity and migration status)

#### Managers

In total, 6944 (69%) HCWs reported feeling supported by a manager at T2. The multilevel binary logistic regression models (table 4) showed no evidence that feeling supported by a manager at T2 varied by ethnicity or migration status.

**Table 4:** Variations in feeling supported by managers and colleagues by ethnicity and migration status

Exposure variables (T1)	Manager support at T2				
	Felt Supported				
	n (%)	OR	95% CI	AOR*	95% CI
Ethnicity†					
White British	5597 (69.41)	–	–	–	–
White other‡	585 (67.83)	0.95	0.77 to 1.16	0.95	0.76 to 1.19
Black§	202 (71.76)	1.09	0.76 to 1.55	0.95	0.63 to 1.41
Asian¶	339 (68.92)	0.97	0.76 to 1.23	0.93	0.74 to 1.16
Mixed/other**	192 (62.24)	0.74	0.42 to 1.30	0.79	0.45 to 1.39
Migration status††					
Born in UK	5942 (68.99)	–	–	–	–
Born in EU (not UK)	391 (63.53)	0.82	0.60 to 1.14	0.85	0.60 to 1.20
Born elsewhere	609 (71.84)	1.15	0.90 to 1.46	1.09	0.80 to 1.49

Exposure variables (T1)	Colleague support at T2				
	Felt supported				
	n (%)	OR	95% CI	AOR*	95% CI
Ethnicity‡‡					
White British	6408 (82.50)	–	–	–	–
White other‡	656 (78.35)	**0.76**	**0.61 to 0.94**	**0.79**	**0.64 to 0.99**
Black§	225 (80.26)	0.84	0.62 to 1.34	0.71	0.50 to 1.03
Asian¶	384 (77.33)	**0.72**	**0.63 to 0.81**	**0.65**	**0.57 to 0.74**
Mixed/other**	216 (76.79)	**0.71**	**0.58 to 0.87**	0.82	0.61 to 1.09
Migration status§§					
Born in UK	6787 (81.96)	–	–	–	–
Born in EU (not UK)	452 (79.24)	0.85	0.71 to 1.02	0.86	0.72 to 1.02
Born elsewhere	679 (78.38)	0.80	0.63 to 1.02	**0.70**	**0.52 to 0.94**

Counts reflect participants with complete data on the exposure and, for adjusted models, complete data on all covariates included in the model.

* Model adjusted for age, sex, role and GHQ-12 score at T2 and reported as AOR.

† n=9613 included in the unadjusted analysis, n=8903 included in the adjusted analysis.

‡ Includes White Irish, White Gypsy or Irish Traveller and any other White background.

§ Includes Black African, Black Caribbean and any other Black background.

¶ Includes Indian, Pakistani, Bangladeshi, Chinese, Arab or any other Asian background.

** Includes any mixed background or other ethnic groups.

†† n=9587 included in the unadjusted analysis, n=8878 included in the adjusted analysis.

‡‡ n=9620 included in the unadjusted analysis, n=8909 included in the adjusted analysis.

§§ n=9594 included in the unadjusted analysis, n=8884 included in the adjusted analysis.

AOR, Adjusted Odds Ratio; EU, European Union; GHQ-12, 12-item General Health Questionnaire.

#### Colleagues

In total, 7924 (81%) HCWs reported feeling supported by colleagues at T2. The multilevel binary logistic regression models (table 4) showed HCWs from the White Other ethnic groups (AOR 0.79; 95% CI 0.64 to 0.99) and HCWs from the Asian ethnic groups (AOR 0.65; 95% CI 0.57 to 0.74) were less likely than HCWs from the White British ethnic group to feel supported by their colleagues at T2 after adjustment. The regression models (table 4) showed HCWs who were born somewhere other than the UK and EU (AOR 0.70; 95% CI 0.52 to 0.94) were less likely than HCWs born in the UK to feel supported by their colleagues at T2 after adjustment.

## Discussion

In this cohort of NHS staff, HCWs from White Other and Asian ethnic groups, and those born outside the UK/EU, were less likely to feel supported by colleagues. In contrast, we found no evidence of disparities in formal support programme use or perceived managerial support by ethnicity or migration status.

### Findings in relation to previous research

A notable finding in this cohort was the high prevalence of probable common mental disorder at 6 months (51% using the GHQ-12 threshold). This aligns with earlier NHS CHECK analyses reporting similarly elevated levels of distress among NHS staff during the first wave of the pandemic.^1^ However, estimates from occupational surveys should be interpreted cautiously. Previous research has shown that the occupation-specific surveys also tend to yield higher prevalence estimates than general population studies.^21^ We have observed patterns consistent with this possibility in related NHS staff analyses, where the rate of probable common mental disorder was 53% using the GHQ-12 but 22% for using a gold-standard diagnostic interview.^22^ Nevertheless, this is still a significant proportion of staff who may require support and therefore reinforces the value in identifying inequity in support access.

Existing evidence shows that HCWs from ethnic minority and migrant groups are more likely to experience abuse at work,^9^ and international nurses and HCWs from ethnic minority groups often feel devalued and experience poorer workplace relationships.^23^ However, far less is known about their access to positive workplace resources such as peer support. This study extends existing literature by demonstrating that inequalities are not limited to negative workplace experiences but also include disparities in perceived colleague support.

In contrast to previous research showing lower engagement with NHS Talking Therapies and staff well-being hubs among Black, Asian and Mixed ethnic groups,^5 24^ we found no evidence of inequities in formal support use; however, descriptive patterns suggested lower uptake of formal mental health support among Black staff (14%) compared with White British staff (22%). Participation from Black and other minority ethnic HCWs was relatively low, which limits statistical power and potentially under-represents those with more negative experiences; therefore, these findings should be interpreted cautiously.

### Strengths and limitations

This study has several limitations.

First, the baseline response rate across participating Trusts was 15%, raising the possibility of response bias. It is possible that those experiencing the most severe distress or workplace discrimination may have been less likely to participate. We applied response weights based on workforce characteristics to improve representativeness; however, weighting cannot account for unmeasured differences between responders and non-responders. Retention between baseline and 6-month follow-up was similar across ethnic and migrant groups (81%–89%), reducing concern that differential attrition substantially biased estimates of association. As our primary aim was to examine relative differences between groups rather than precise prevalence estimates, associations may be less sensitive to overall response rates unless non-response differed systematically by both exposure and outcome. Nevertheless, findings should be interpreted cautiously.

Second, small numbers within some ethnic minority groups required collapsing categories, potentially masking important within-group heterogeneity. Minority ethnic HCWs were under-represented relative to the NHS workforce, a challenge common in health research.^25^ Intersectional analyses (eg, ethnicity by migration status) were not feasible due to limited power. Future studies should prioritise strategies to improve participation from these groups.

Third, support use was analysed as a binary outcome (used vs not used), combining diverse forms of practical and psychological support. While this allowed sufficient power for regression analyses, it may obscure differences in uptake of specific services. Descriptive statistics were provided for individual support types, but small numbers precluded inferential testing. Finally, survey questions did not capture timing of support use, limiting temporal interpretation.

Despite these limitations, the study used a large, multisite NHS sample including clinical and non-clinical staff, and applied workforce-based weighting to enhance representativeness.

### Implications

Given the high rates of psychological distress in this cohort, our finding of broadly equitable formal support use alongside disparities in perceived colleague support suggests that service availability alone may be insufficient: addressing antecedent workplace conditions (eg, workload, staffing, discrimination, team climate, psychological safety) is likely necessary both to reduce distress and to enable informal support to function as a protective resource. Although these data were collected during the COVID-19 pandemic, this context arguably magnified rather than created existing systemic pressures within the NHS, including workforce shortages, retention difficulties^26^,^28^ and racialised inequalities. Additionally, racial hostilities towards migrants are still prevalent, as demonstrated by the far-right riots in the UK in 2024^29^; this led to the British Medical Association putting out guidance on how to support colleagues who had been affected.^30^ Examining support under conditions of extreme demand, therefore, offers insight into how resilient and equitable workplace support structures are when strained and provides lessons for strengthening inclusive support in the current and future NHS.

Beyond workplace conditions, there are interventions which attempt to address colleague and managerial support. Evidence shows active listening training increases managers’ confidence to support staff,^31^ and well-being conversations in one NHS clinical commissioning group were associated with sickness absence reductions from three per cent to less than one per cent within 4 months.^32^ However, the overall evidence for such training is limited and restricted to small case studies.

Our findings also indicate the value of disaggregating White ethnic subgroups. The latest NHS Workforce Race Equality Standard report found HCWs from White Gypsy or Irish Traveller ethnic groups experienced the highest level of staff abuse.^33^ Although very few were included in our sample, and so this may not explain our finding, it does highlight that further disaggregation of the ‘White Other’ group is valuable. The NHS Workforce Race Equality Standard has previously not distinguished between White British HCWs and those who would be in our defined ‘White Other’ ethnic group, such as individuals born in Europe. Given our findings, we encourage the NHS Workforce Race Equality Standard to continue detailed analysis of White ethnic sub-groups to better understand these disparities.

## Conclusions

In summary, this study found equitable use of formal support programmes and managerial support, but clear disparities in colleague support by ethnicity and migration status. Given the protective impact that colleague support can have against crises like the COVID-19 pandemic and ongoing systemic issues in the NHS. Future research should focus on furthering our understanding of the mechanisms driving workplace disparities for HCWs from ethnic minority and migrant backgrounds.

## Supplementary material

10.1136/oemed-2025-110203 online supplemental file 1

## Data Availability

Data are available on reasonable request.
