# Supplementary material for: Inequalities in NHS staff support among those from ethnic minority and migrant groups during the COVID-19 pandemic
Source: Occup Environ Med. 2026 Apr 7;83(1):e110203. doi: 10.1136/oemed-2025-110203 (PMC13151436; doi:10.1136/oemed-2025-110203)
Supplement: online supplemental file 1 [file oemed-83-1-s001.docx]

**Supplementary Material 1 – NHS CHECK Consent Form and Participant Information Sheet**

**Supplementary Material 2.**

Table 2.1.

Participants in the NHS Check Study by NHS Trust

| Participating NHS Trusts | No. of participants | | ~ % of workforce participated |
| --- | --- | --- | --- |
| Avon and Wiltshire Mental Health Partnership NHS Trust | | 979 | 22.6% |
| Cornwall Partnership NHS Foundation Trust | | 1093 | 27.5% |
| Cambridgeshire and Peterborough NHS Foundation Trust | | 1495 | 35.3% |
| Cambridge University Hospitals NHS Foundation Trust | | 1017 | 9.0% |
| Devon Partnership NHS Trust | | 1386 | 42.2% |
| East Suffolk and North Essex NHS Foundation Trust | | 447 | 4.4% |
| Gloucestershire Hospitals NHS Foundation Trust | | 731 | 8.6% |
| Guys and St Thomas' NHS Foundation Trust | | 3044 | 15.4% |
| King's College Hospital NHS Foundation Trust | | 2053 | 15.8% |
| Lancashire and South Cumbria NHS Foundation Trust | | 852 | 13.4% |
| Norfolk and Norwich University Hospitals NHS Foundation | | 1376 | 13.1% |
| Nottinghamshire Healthcare NHS Foundation Trust | | 2522 | 28.5% |
| Royal Papworth Hospital NHS Foundation Trust | | 153 | 7.3% |
| Sheffield Health and Social Care Trust | | 485 | 18.6% |
| South London and Maudsley NHS Foundation Trust | | 1550 | 30.1% |
| Tees Esk and Wear Valleys NHS Foundation Trust | | 1980 | 27.1% |
| University Hospitals of Derby and Burton NHS | | 503 | 3.8% |
| University Hospitals of Leicester NHS Foundation Trust | | 1680 | 10.1% |
|  | |  |  |
| Total | | **23, 346** |  |

*Note. This table is up to date as of 07.12.21 and may fluctuate depending on the addition and withdrawal of participants.*

**Supplementary Material 3.**

Table 3.1.

Summary of NHS CHECK Surveys used in this analysis by time point.

| **Survey** | **Timepoint (T)** | **Launched** | **Closed** | **Key milestones in COVID-19 pandemic in UK** |
| --- | --- | --- | --- | --- |
| Baseline | 1 | April 2020 | January 2021 | 5 weeks after initial lockdown |
| Six-month follow-up^a^ | 2 | October 2020 | August 2021 | UK enters second national lockdown (Nov 2020) |
| *Note. The survey launch date was fixed, but the start date for each NHS Trust was staggered based on their agreement to participate.*  *^a^Individuals were contacted with survey invites six months after they completed the baseline survey.* | | | | |

**Supplementary Material 4**

Table 4.1.

The proportion of HCWs included in the analysis by the original 17 ethnic groups

| **Ethnic Group** | **Frequency (n)** | **Weighted (%)** |
| --- | --- | --- |
| White English/Welsh/Scottish/Northern Irish | 7814 | 75.00 |
| White Irish | 183 | 1.67 |
| White Gypsy or Irish Traveller | * | <1 |
| Any other White background | 649 | 6.12 |
| Mixed White and Black Caribbean | 53 | <1 |
| Mixed White and Black African | 30 | <1 |
| Mixed White and Asian | 64 | <1 |
| Any other Mixed/Multiple ethnic background | 77 | <1 |
| Indian | 240 | 4.59 |
| Pakistani | 44 | <1 |
| Bangladeshi | 27 | <1 |
| Chinese | 46 | <1 |
| Any other Asian background | 133 | 2.73 |
| Black African | 149 | 2.64 |
| Black Caribbean | 112 | 1.98 |
| Any other Black background | 17 | <1 |
| Arab | 13 | <1 |
| Any other ethnic group | 61 | 1.48 |
| **Total** | 9716 | 100 |

**Supplementary Material 5.**

Multi-level generalised linear models showed that HCWs who used support had an estimated 0.35 unit increase in their GHQ-12 score at T2 compared to those who did not use support at T2 (OR 0.35; 95% CI 0.24 to 0.46). Secondly, HCWs who felt supported by their manager at T2 had a 1.13 unit decrease in their GHQ-12 total score at T2 compared to those who did not feel supported (OR -1.13; 95% CI -1.41 to -0.86). Finally, HCWs who felt supported by their colleagues at T2 had a 1.03 unit decrease in their GHQ-12 total score at T2 compared to those who did not feel supported at T2 (OR -1.03; 95% CI -1.21 to -0.85).

Table 5.1.

Associations between support use, manager support and colleague support and score on the GHQ-12.

| **Model** | **Coefficient** | **P-Value** | **95% CI** |
| --- | --- | --- | --- |
| **Support Used** | 0.35 | <0.001 | 0.24 - 0.46 |
| **Manager Support** | -1.13 | <0.001 | -1.41 - -0.86 |
| **Colleague Support** | -1.03 | <0.001 | -1.21 - -0.85 |

**Supplementary Material 6**

*Table 6.1.*

*Median GHQ-12 scores for HCWs with probable CMD at T2 by ethnicity and migration status*

|  | **Probable CMD** ^ab^ **at T2** | |  |
| --- | --- | --- | --- |
|  | **n (%)** | **Median** | **Interquartile range** |
| **Total sample** | 4817 (51.335) | 4 | 1-8 |
| **Ethnicity** | | |  |
| White British | 3829 (52.49) | 4 | 1-8 |
| White Other | 444 (51.93) | 4 | 1-8 |
| Black | 117 (53.84) | 3 | 1-6 |
| Asian | 242 (40.75) | 3 | 1-8 |
| Mixed/Other | 156 (65.18) | 4 | 1-8 |
| **Migration status** | | |  |
| Born in UK | 4075 (52.33) | 4 | 1-8 |
| Born in EU (Not UK) | 305 (53.84) | 4 | 1-8 |
| Born elsewhere | 398 (44.24) | 3 | 1-6 |
| *^a^ Common Mental Disorder*  *^b^ (GHQ-12: cut-off ≥4, scale range: 0–12)*  *^c^ includes White Irish, White Gypsy or Irish Traveller and any other White background*  *^d^ Includes Black African, Black Caribbean and any other Black background*  *^e^ Includes Indian, Pakistani, Bangladeshi, Chinese, Arab or any other Asian background*  *^f^ Includes any mixed background or other ethnic groups* | | | |

**Supplementary Material 7**

Table 7.1.

Proportion of Staff Born in UK or elsewhere by Ethnicity

| **Ethnicity** | **Born in UK**  **n (%)** | **Born outside of UK^a^**  **n (%)** | ***Total***  ***n (%)*** |
| --- | --- | --- | --- |
| White British | 7561 (96.87) | 216 (3.13) | *777 (100)* |
| White Other | 178 (20.78) | 657 (79.22) | *835 (100)* |
| Black | 142 (48.70) | 136 (51.30) | *278 (100)* |
| Asian | 185 (33.65) | 316 (66.35) | *501 (100)* |
| Mixed/Other | 190 (52.56) | 95 (47.44) | *285 (100)* |
| ***Total***  ***n (%)*** | *8256 (81.24)* | *1420 (18.76)* | *9676* |

^a^These categories were combined due to small cell counts.

**Supplementary Material 8.**

Table 8.1. Support type use by ethnicity.

| Support type (T2) | | White British  n(%) | White Other  n(%) | Black  n(%) | Asian  n(%) | Mixed/Other  n(%) |
| --- | --- | --- | --- | --- | --- | --- |
| Formal Mental Health Support | Did not use | 2932 (31.35%) | 344 (38.08%) | 141 (47.09%) | 197 (30.69%) | 112 (38.76%) |
|  | Used | 1749 (22.35%) | 219 (25.13%) | 36 (13.98%) | 87 (14.20%) | 70 (16.21%) |
|  | Missing | 3133 (46.31%) | 273 (36.79%) | 101 (38.93%) | 219 (55.11%) | 103 (45.03%) |
| Employee Assistance Programmes | Did not use | 2563 (30.33%) | 307 (34.04%) | 95 (34.61%) | 138 (25.90%) | 95 (31.91%) |
|  | Used | 478 (5.65%) | 28 (2.99%) | 20 (6.61%) | 30 (4.42%) | 14 (4.05%) |
|  | Missing | 4773 (64.02%) | 501 (62.98%) | 163 (58.78%) | 335 (69.68%) | 176 (64.05%) |
| Occupational Health | Did not use | 4842 (63.62%) | 498 (61.27%) | 135 (48.48%) | 274 (59.52%) | 169 (52.49%) |
|  | Used | 1575 (20.48%) | 167 (21.17%) | 87 (31.54%) | 128 (21.26%) | 73 (33.13%) |
|  | Missing | 1397 (15.90%) | 171 (17.56%) | 56 (19.98%) | 101 (19.22%) | 43 (14.38%) |
| Practical Support | Did not use | 4497 (60.31%) | 539 (66.01%) | 146 (56.98%) | 288 (58.97%) | 154 (61.03%) |
|  | Used | 2283 (27.91%) | 202 (22.26%) | 75 (25.71%) | 137 (26.35%) | 82 (25.02%) |
|  | Missing | 1034 (11.78%) | 95 (11.73%) | 57 (17.31%) | 78 (14.67%) | 49 (13.94%) |
| Wellbeing Support | Did not use | 6754 (85.38%) | 703 (83.67%) | 230 (83.90%) | 409 (82.13%) | 242 (84.06%) |
|  | Used | 639 (9.19%) | 79 (9.91%) | 25 (7.99%) | 51 (8.61%) | 23 (8.95%) |
|  | Missing | 421 (5.43%) | 54 (6.42%) | 23 (8.10%) | 43 (9.26%) | 20 (6.99%) |
| Workplace Support | Did not use | 4017 (55.50%) | 447 (55.25%) | 132 (52.60%) | 262 (55.25%) | 147 (56.29%) |
|  | Used | 835 (9.56%) | 116 (12.11%) | 48 (14.57%) | 51 (11.04%) | 28 (15.55%) |
|  | Missing | 2962 (34.94%) | 273 (32.64%) | 98 (32.83%) | 190 (33.71%) | 110 (28.15%) |
| Information Support | Did not use | 3584 (51.80%) | 405 (52.62%) | 118 (45.67%) | 250 (53.91%) | 136 (56.31%) |
|  | Used | 1808 (24.40%) | 219 (23.65%) | 63 (23.40%) | 119 (22.11%) | 54 (23.40%) |
|  | Missing | 2422 (23.80%) | 212 (23.73%) | 97 (30.93%) | 134 (23.98%) | 95 (20.29%) |

Note. Categories are not mutually exclusive; participants could report use of more than one support type.

Table 8.2. Support type use by migration status.

|  | | Birth Country | | |
| --- | --- | --- | --- | --- |
| Support Type (T2) | | United Kingdom | EU (not UK) | Other (non-EU) |
| Formal Mental Health Support | Did not use | 3098 (31.80%) | 243 (38.57%) | 377 (36.83%) |
|  | Used | 1853 (21.92%) | 155 (27.64%) | 146 (13.85%) |
|  | Missing | 3318 (46.28%) | 175 (33.79%) | 326 (49.32%) |
| Employee Assistance Programmes | Did not use | 2726 (30.55%) | 192 (31.37%) | 271 (29.33%) |
|  | Used | 501 (5.44%) | 15 (3.18%) | 53 (5.53%) |
|  | Missing | 5042 (64.01%) | 366 (65.45%) | 525 (65.14%) |
| Occupational Health | Did not use | 5095 (63.00%) | 337 (60.08%) | 474 (56.82%) |
|  | Used | 1690 (20.88%) | 121 (22.37%) | 211 (24.55%) |
|  | Missing | 1484 (16.12%) | 115 (17.55%) | 164 (18.63%) |
| Practical Support | Did not use | 4730 (60.20%) | 362 (64.12%) | 516 (60.60%) |
|  | Used | 2451 (28.02%) | 130 (21.63%) | 193 (23.94%) |
|  | Missing | 1088 (11.78%) | 81 (14.26%) | 140 (15.46%) |
| Wellbeing Support | Did not use | 7124 (85.07%) | 476 (83.89%) | 717 (83.64%) |
|  | Used | 679 (9.24%) | 58 (9.67%) | 75 (8.11%) |
|  | Missing | 466 (5.69%) | 39 (6.44%) | 57 (8.25%) |
| Workplace Support | Did not use | 4240 (55.27%) | 304 (55.59%) | 444 (55.17%) |
|  | Used | 883 (9.69%) | 80 (11.69%) | 109 (13.47%) |
|  | Missing | 3146 (35.03%) | 189 (32.72%) | 296 (31.36%) |
| Information Support | Did not use | 3785 (51.71%) | 274 (52.17%) | 417 (52.40%) |
|  | Used | 1910 (24.09%) | 146 (24.62%) | 202 (23.74%) |
|  | Missing | 2574 (24.20%) | 153 (23.21%) | 230 (23.86%) |

Note. Categories are not mutually exclusive; participants could report use of more than one support type.
